# Pathogenicity, Transmission and Antigenic Variation of H5N1 Highly Pathogenic Avian Influenza Viruses

**DOI:** 10.3389/fmicb.2016.00635

**Published:** 2016-05-06

**Authors:** Peirong Jiao, Hui Song, Xiaoke Liu, Yafen Song, Jin Cui, Siyu Wu, Jiaqi Ye, Nanan Qu, Tiemin Zhang, Ming Liao

**Affiliations:** ^1^National and Regional Joint Engineering Laboratory for Medicament of Zoonosis Prevention and ControlGuangzhou, China; ^2^Key Laboratory of Animal Vaccine Development, Ministry of AgricultureGuangzhou, China; ^3^Key Laboratory of Zoonosis Prevention and Control of GuangdongGuangzhou, China; ^4^College of Veterinary Medicine, South China Agricultural UniversityGuangzhou, China; ^5^Pulike Biological Engineering Inc.Luoyang, China; ^6^College of Engineering, South China Agricultural UniversityGuangzhou, China

**Keywords:** H5N1, pathogenicity, transmissibility, antigenic variation, vaccine

## Abstract

H5N1 highly pathogenic avian influenza (HPAI) was one of the most important avian diseases in poultry production of China, especially in Guangdong province. In recent years, new H5N1 highly pathogenic avian influenza viruses (HPAIV) still emerged constantly, although all poultry in China were immunized with H5N1 vaccinations compulsorily. To better understand the pathogenicity and transmission of dominant clades of the H5N1 HPAIVs in chicken from Guangdong in 2012, we chose a clade 7.2 avian influenza virus named A/Chicken/China/G2/2012(H5N1) (G2) and a clade 2.3.2.1 avian influenza virus named A/Duck/China/G3/2012(H5N1) (G3) in our study. Our results showed that the chickens inoculated with 10^3^ EID_50_ of G2 or G3 viruses all died, and the titers of virus replication detected in several visceral organs were high but different. In the naive contact groups, virus shedding was not detected in G2 group and all chickens survived, but virus shedding was detected in G3 group and all chickens died. These results showed that the two clades of H5N1 HPAIVs had high pathogenicity in chickens and the contact transmission of them was different in chickens. The results of cross reactive HI assay showed that antigens of G2 and G3 were very different from those of current commercial vaccines isolates (Re-4, Re-6, and D7). And to evaluate the protective efficacy of three vaccines against most isolates form Guangdong belonging to clade 2.3.2.1 in 2012, G3 was chosen to challenge the three vaccines such as Re-4, Re-6, and D7. First, chickens were immunized with 0.3 ml Re-4, Re-6, and D7 inactivated vaccines by intramuscular injection, respectively, and then challenged with 10^6^ EID_50_ of G3 on day 28 post-vaccination. The D7 vaccine had 100% protection against G3 for chickens, the Re-6 vaccine had 88.9%, and the Re-4 vaccine only had 66.7%. Our results suggested that the D7 vaccine could prevent and control H5N1 virus outbreaks more effectively in Guangdong. From the above, it was necessary to conduct continuously epidemiological survey and study the pathogenicity and antigenic variation of avian influenza in Southern China.

## Introduction

Influenza A viruses are the most important pathogen of three types (A, B, and C) of influenza viruses, to both the poultry industry and human health. To date, avian influenza viruses representing 16 HA and 9 NA subtypes have been detected in wild birds and poultry throughout the world (Webster et al., 1992; Fouchier and Munster, 2009). According to the virulence of viruses, avian influenza viruses are divided into highly pathogenic avian influenza virus (HPAIV), low pathogenic avian influenza virus (LPAIV), and non-pathogenic avian influenza virus (NPAIV). However, only some influenza viruses of H5 and H7 subtype are highly pathogenic to poultry.

The H5N1 HPAIV was first isolated in Guangdong, China in 1996 (Xu et al., 1999). In 1997, H5N1 HPAIVs had repeatedly caused serious outbreaks among poultry farms and markets in Hong Kong, which resulted in heavy losses. And it was the first report that H5N1 HPAIV infected human in “Hong Kong Flu” in 1997, causing six deaths in 18 infection cases (Claas et al., 1998; Subbarao et al., 1998). In 2002, a new H5N1 HPAI outbreak in Hong Kong infected millions of birds, including several types of wild water fowl. This was the first time the H5N1 HPAIV was found to infect water fowl (Lee et al., 2005; Nguyen et al., 2005). Between 2003 and 2005, the H5N1 HPAI repetitively broke out in East Asia and South Asia, and even spread to Europe and Africa. This resulted in more than 150 million birds dead or slaughtered and 53 human fatalities (Sturm-Ramirez et al., 2004; World Health Organization, 2005). Since 2003, the H5N1 HPAI had continued influencing more than 60 states or areas including Laos, Vietnam, Thailand, Hong Kong, and China (World Health Organization, 2013b). From 2003 to 1 May 2015, 840 laboratory-confirmed human cases of H5N1 HPAIVs infection were officially reported to WHO from 16 countries; of these cases, 447 died (World Health Organization, 2015). Consequently, H5N1 HPAIVs are zoonotic etiological agents recognized as a severe threat to both the poultry industry and human public health around the world.

In terms of antigenic characteristics, H5N1 HPAIVs were divided into 10 clades (0–9) and numerous subclades by World Health Organization/World Organization for Animal Health/Food and Agriculture Organization H5N1 Evolution Working Group (2008)^1^. Complicated breeding environments, the long distance transport of live poultry, and wild bird migration resulted in all known clades circulating endlessly in poultry in China. Especially during 2005 and 2006, H5N1 viruses of clades 2.2, 2.3.2, 2.3.4, 4, 7, and 9 circulated all over China. Since 2007, viruses of clades 2.3.2, 2.3.4, and 7 have predominantly co-circulated continuously in domestic poultry and waterfowl in China (Smith et al., 2009; Jiang et al., 2010; Li et al., 2010). Later, several studies results showed that the pathogenicity of clade 2.3.2 viruses were intensifying in aquatic birds (Sakoda et al., 2010). Viruses of clade 7 began spreading in chickens across the northern of China in 2005, which had a high pathogenicity in chickens, but only a few viruses were isolated from aquatic birds. In 2008, H5N1 HPAI caused clade 7.2 viruses broke out in several cities in North China and caused a considerable amount of deaths in poultry. In 2010, a new H5N1 HPAIV belonging to clade 2.3.2.1 was isolated from South Asia, and 48 humans were reported to have been infected with the virus (Reid et al., 2011). In 2011, the H5N1 HPAI caused clade 2.3.2.1 viruses broke out in crows (World Health Organization, 2012). From 2012 to 2013, the H5N1 HPAIVs belonging to 2.3.2.1, 2.3.4, and 7.2 clades were detected in birds and/or environmental samples in China (World Health Organization, 2013a), but the most isolates belonged to clade 2.3.2.1. The pathogenicity of different clades varied in poultry and wild birds, but the movement and interaction of H5N1 viruses between them was still not clear until now.

To better understand the pathogenicity and transmissibility of different clade of H5N1 isolates from poultry in Guangdong in 2012, we selected two viruses—A/Chicken/China/G2/2012(H5N1) (G2) and A/Duck/China/G3/2012(H5N1) (G3)—to carry out their infection experiments. To evaluate the antigenic variation of these viruses and protective efficacy of current commercial vaccines against most isolates from Guangdong in 2012, G3 (belonging to clade 2.3.2.1) was chosen to challenge three commercial vaccines such as Re-4, Re-6, and D7.

## Materials and methods

### H5N1 HPAIV variants and propagation

The two H5N1 HPAIVs—A/Chicken/China/G2/2012(H5N1) (G2) and A/Duck/China/G3/2012(H5N1) (G3)—used in this study were isolated from cloacal swabs of apparently healthy birds in live bird markets during 2012. They were purified and propagated by three rounds of limiting dilution in the allantoic cavity of 9–11 days old specific-pathogen-free (SPF) embryonated chicken eggs (Jiao et al., 2014; Yuan et al., 2014). The allantoic fluid from multiple eggs was pooled, clarified by centrifugation, and frozen in aliquots at −70°C. The G2 and G3 inactivated antigens and positive serums were provided by College of Veterinary Medicine, South China Agricultural University. The 50% egg infectious dose (EID_50_) was calculated according to the method published by Reed and Muench (1938) using the serial titration of eggs. All experiments were carried out in Animal Biosafety Level 3 (ABSL-3) facilities.

### Genetic and phylogenetic analyses

The viral RNA was extracted from the allantoic fluid supernatant using Trizol LS Reagent (Life Technologies, Inc.). A reverse transcription polymerase chain reaction (RT-PCR) was conducted using Superscript III (Invitrogen, Carlsbad, CA, USA) and Uni12 (5-AGCAAAAGCAGG-3) primer. Eight genes were amplified using universal primers (Hoffmann et al., 2001), and the PCR products were purified using the mini PCR Purification Kit (Promega). Sequencing was performed by Shanghai Invitrogen Biotechnology Co., Ltd. The sequencing data were compiled with the Seqman program of Lasergene 7 (DNASTAR, Inc.). Amino acid sequence similarities were identified with the Lasergene 7 Megalign program (DNASTAR). The hemagglutinin (HA) gene phylogenetic tree of the H5N1 HPAIVs was created with MEGA 5 software (Sinauer Associates, Inc., Sunderland, MA).

The nucleotide sequences of A/Chicken/China/G2/2012(H5N1) (G2) and A/Duck/China/G3/2012(H5N1) (G3) were available from GenBank under the accession numbers KU851866-KU851867.

### Pathogenicity and transmission

Five-week-old SPF White Leghorn chickens were purchased from Beijing Merial Vital Laboratory Animal Technologies Co., LTD, Beijing, China.

To determine the pathogenicity and transmission of the two H5N1 HPAIVs, twenty-seven chickens were equally divided into three groups G2, G3, and control. Six chickens of G2 and G3 group were inoculated intranasally with 10^3^ EID_50_ of G2 or G3 viruses, respectively; the other three chickens of each group were inoculated intranasally with the same volume of phosphate buffered saline (PBS), as naive contact housed with the inoculated chickens. The chickens of control group were inoculated intranasally with the same volume of PBS. All chickens were observed for clinical symptoms for 14 days. Three inoculated chickens in each group were euthanized at 3 days post-inoculation (DPI), and the lungs, kidneys, liver, heart, spleen, and brain were collected. Similar executions were performed on chickens that died during the observation. Oropharyngeal and cloacal swabs were collected from all chickens at 3, 5, 7, 9, and 11 DPI, and suspended in 1 ml isolation media PBS (pH 7.4). All of the tissues and swabs were collected and titrated for virus infectivity in eggs, as described previously (Chen et al., 2004; Jiao et al., 2008). Seroconversion of the surviving chickens on 14 DPI was confirmed by hemagglutinin inhibition (HI) test. HI titers of the serums were detected using 1% chicken red blood cells by a standard method (Takatsy and Barb, 1973). All animal experiments were conducted under the guidance of SCAU's Institutional Animal Care and Use Committee. Our animal experiments in this study had been approved by SCAU and were carried out in high-efficiency particulate air-filtered isolators (size: 2200 × 850 × 1700 mm) and ABSL-3 facilities.

### Vaccine-challenge

To evaluate the antigenic variation of these viruses and protective efficacy of current commercial vaccines against most isolates from Guangdong in 2012, 3-week-old SPF White Leghorn chickens were purchased from Beijing Merial Vital Laboratory Animal Technologies Co., LTD, Beijing, China. Re-4 and Re-6 vaccines strain inactivated antigens, positive serums, and vaccines were purchased from Weike Biotechnology Co., Ltd., Harbin, China. D7 (H5N2) vaccines strain inactivated antigens, positive serums, and vaccines were purchased from Guangzhou South China Biological Medicine Co. Ltd., Guangdong, China.

Thirty-six chickens were divided into four groups (*n* = 9), and three groups were immunized with 0.3 ml of Re-4, Re-6, or D7 inactivated vaccines via intramuscular injection, the control group received 0.3 ml of PBS intramuscularly. Serum was collected from every chicken on 14 and 28 day-post-vaccination (DPV) for HI titers determination.

At 28 DPV, chickens were intranasally challenged with 200ul 10^6^EID_50_ of A/Duck/China/G3/2012(H5N1) (G3). Oropharyngeal and cloacal swabs were taken on days 3, 5, 7, 9, and 11 post-challenge, including chickens that died during this period. All swabs were immediately suspended in 1 ml isolation media PBS, which were inoculated into 9–10 days old embryonated chicken eggs for examination of virus shedding. All surviving chickens were observed for clinical symptoms for 14 days and collected serum for seroconversion detection in the end.

## Results

### Genetic and phylogenetic analysis

**Figure 1:**
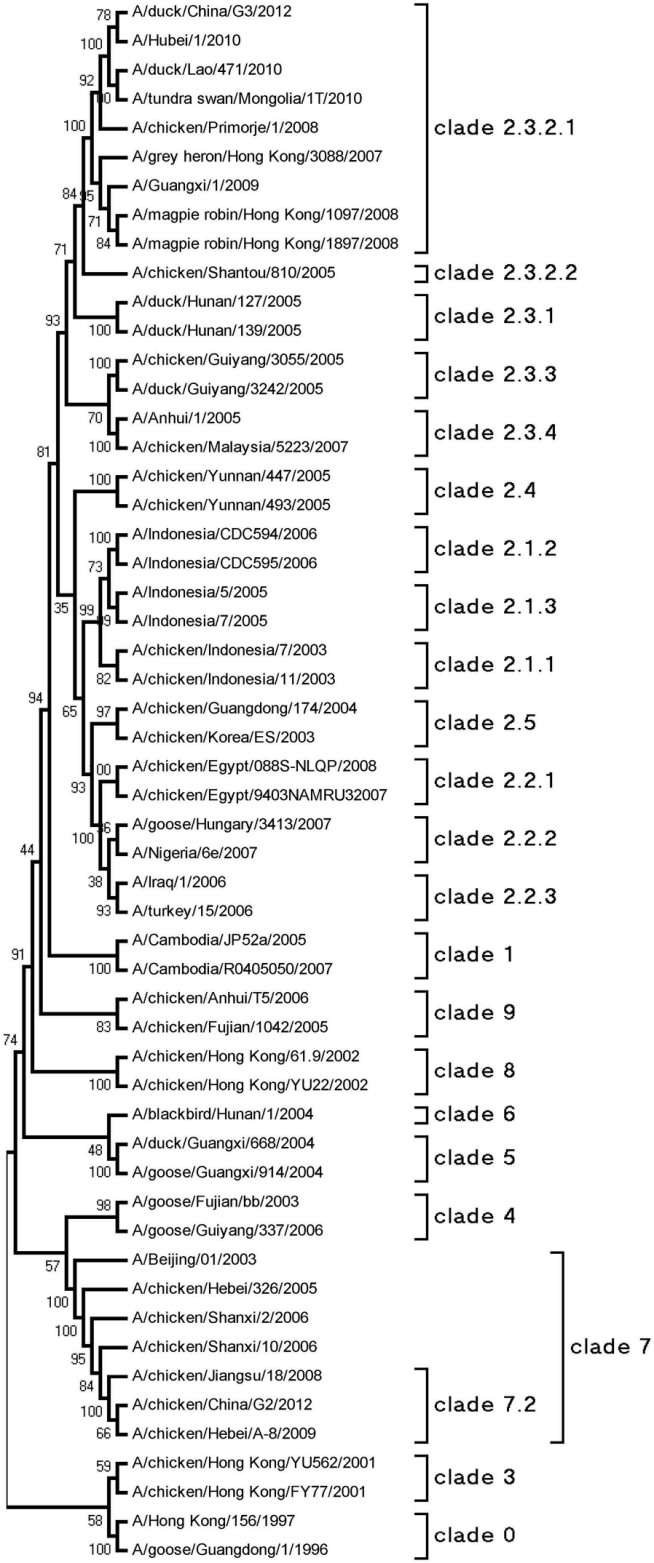
**Phylogenetic analysis of HA**. The trees were constructed by using the neighbor joining method with the Maximum Composite Likelihood model and MEGA5 software with 1000 bootstrap replicates based on the following sequences: HA (A), nucleotides (nt) 29–1732.

The HA genes of each virus were sequenced to determine the molecular evolution of the two viruses. The sequences were compared with representative H5N1 sequences obtained from GenBank. According to antigenic characteristics by the WHO, the HA gene of G2 belonged to clade 7.2, and that of G3 belonged to clade 2.3.2.1 (Figure 1). Their HA genes had a series of basic amino acids at the cleavage site of the HA (-RRRKR/GLF-), which represents the high pathogenicity of the H5N1 AIVs in poultry (Gohrbandt et al., 2011).

The amino acid sequences of the two viruses revealed five conservative potential N-linked glycosylation sites in HA (26, 27, 39, 499, and 558): three in HA1 (26, 27, and 39) and two in HA2 (499 and 558). In addition, the G2 virus lost two potential N-linked glycosylation sites in 178 (NNT) and 209 (NPT), and amino acids at 155 the glycosylation site changed from NSS to NPS (**Table 4**). The G3 HA lost three potential N-linked glycosylation sites in 169 (NNT), 209 (NPT) and 251 (NDT), and amino acids at 301 the glycosylation site changed from NSS to NYS (Table 1).

**Table 1:** **Cleavage site and potential glycosylation sites in HA of the two H5N1 HPAIVs (A/Chicken/China/G2/2012(H5N1) = G2 and A/Duck/China/G3/2012(H5N1) = G3)**.

**Strains**	**Cleavage site**	**potential glycosylation sites**										
	**342-347 -RRRKR/G-**	**26 NNS**	**27 NST**	**39 NVT**	**155 NSS**	**169 NNT**	**178 NNT**	**209 NLT**	**251 NDT**	**301 NSS**	**499 NGT**	**558 NGS**
G2	+^a^	+	+	+	NPS	+	−^b^	−	+	+	+	+
G3	+	+	+	+	+	−	+	−	−	NYS	+	+

a *The “+” means the amino acid sequences of glycosylation sites are same with list above*.

b *The “−” means the glycosylation sites are lost*.

### Pathogenicity of H5N1 HPAIVs in chickens

**Figure 2:**
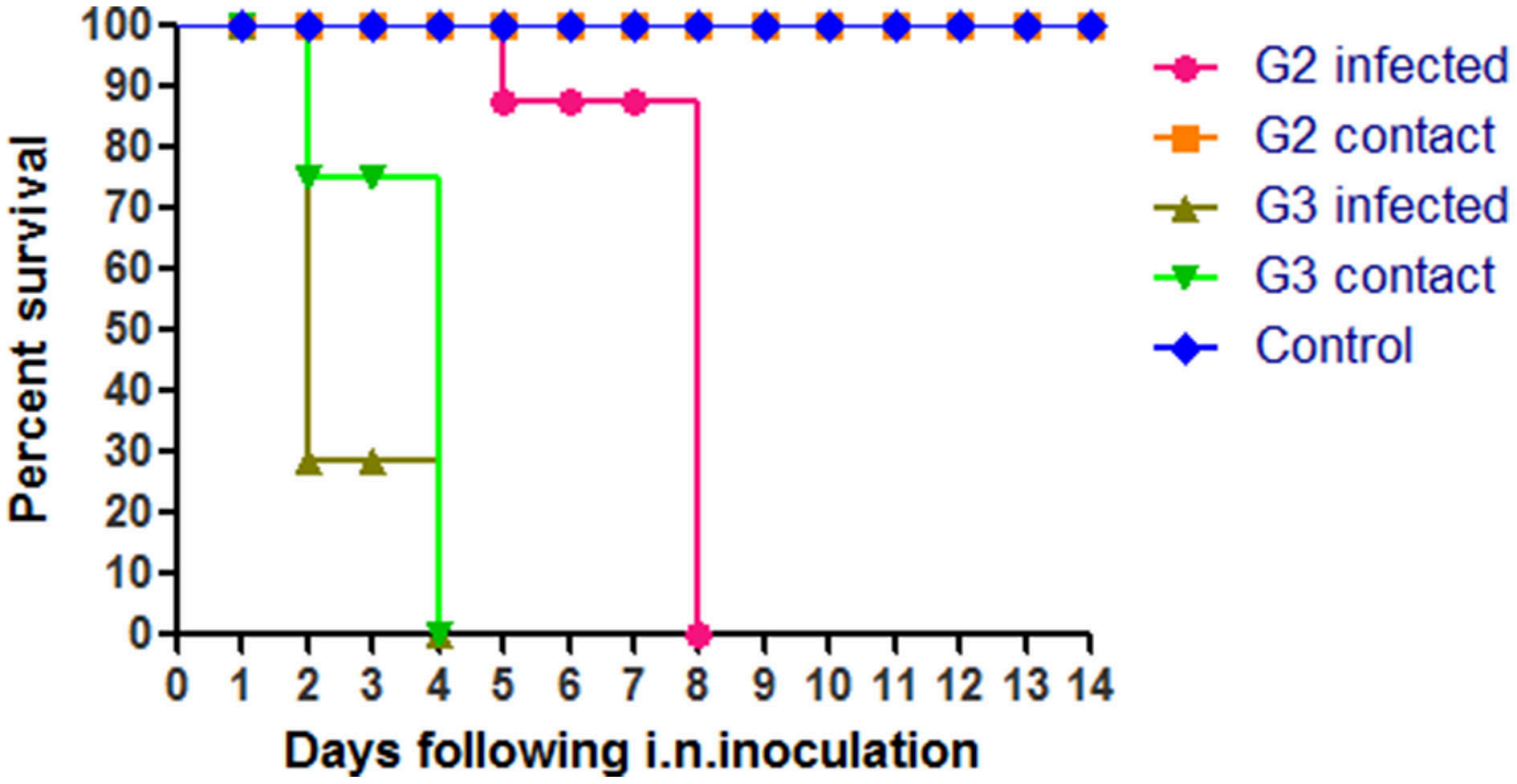
**Lethality of the G2 and G3 viruses in SPF chickens**. The G2 infected chickens were inoculated intranasally with 100μl 10^3^EID_50_ G2 viruses and the G2 contact chickens were housed with them without inoculate. The G3 infected chickens were inoculated intranasally with 100 ul 10^3^EID_50_ G3 viruses and the G3 contact chickens were housed with them without inoculate.

To evaluate the pathogenicity of the two H5N1 HPAIVs, six chickens of each group were inoculated intranasally with 100 μl 10^3^ EID_50_ G2, G3, or PBS, respectively. All chickens in the G3 group began to show clinical typical symptoms as early as two DPI, and were dead by four DPI (Figure 2). However, the inoculated chickens of the G2 group showed clinical signs by four DPI, and all died by eight DPI (Figure 2). So the lethality of G2 and G3 viruses in chickens was 100% (Table 2).

**Table 2:** **Clinical situations and lethality of chickens after inoculated intranasally with the two H5N1 HPAIVs (A/Chicken/China/G2/2012 (H5N1) = G2 and A/Duck/China/G3/2012(H5N1) = G3)**.

**Strains**		**Clinical symptom rates**	**Virus shedding proportions**	**Survival**
G2	Infected^a^	100%	100%	0
	Contact^b^	100%	0	100%
G3	Infected	100%	100%	0
	Contact	100%	100%	0
Control		0	0	100%

a *Chickens inoculated with virus*.

b *Contact chickens housed with those inoculated*.

Eyelid edema, insensibility, diminished appetite and thirst, roughened hair coats, comb cyanosis, torticollis, ataxia, and other neurological symptoms were observed among dead chickens infected with G2 and G3. At necropsy of chickens dead from infection we found slight petechial hemorrhaging in subcutaneous fat, hyperaemia, haemorrhagia, and nig-necrosis in the lungs; hepatomegaly and an amber liver; and hyperaemia and haemorrhagia in the stomachus glandularis. In short, both G2 and G3 viruses produced apparent clinical symptoms and typical pathological changes in severe infected chickens.

To evaluate the replication of the two viruses in chickens, three inoculated chickens in each group were euthanized at three DPI, and the lungs, kidneys, liver, heart, spleen, and brain were collected. Oropharyngeal and cloacal swabs were collected from chickens of each group at 3, 5, 7, 9, and 11 DPI. All of the tissues and swabs were collected and titrated for virus infectivity. In G2 inoculated chickens, the virus replicated in all tested organs on three DPI and the mean titers were 4.33 log EID_50_ in the heart, 2.92 log EID_50_ in the liver, 2.67 log EID_50_ in the spleen, 1.58 log EID_50_ in the lungs, 3.67 log EID_50_ in the kidneys, and 3.25 log EID_50_ in the brain (Table 3). In G3 virus inoculated chickens, the virus replicated to higher and the mean titers were 5.25 log EID_50_ in the liver and 5.5 log EID_50_ in the heart, spleen, lungs, kidneys, and brain, respectively (Table 3). Above all, the replication of G3 in chicken was much higher than that of G2.

**Table 3:** **Replication of the two H5N1 HPAIVs (A/Chicken/China/G2/2012(H5N1) = G2 and A/Duck/China/G3/2012(H5N1) = G3) in SPF chickens^a^**.

**Strains**		**Virus titers in SPF chickens at 3 DPI (log**_**10**_**EID**_**50**_**/0.1ml) in**^**b**^					
		**Heart**	**Liver**	**Spleen**	**Lungs**	**Kidneys**	**Brain**
G2	Infected	4.33 ± 0.52	2.92 ± 0.52	2.67 ± 0.14	1.58 ± 0.14	3.67 ± 0.52	3.25 ± 0.5
	Contact	0	0	0	0	0	0
G3	Infected	5.5 ± 0	5.25 ± 0.43	5.5 ± 0	5.5 ± 0	5.5 ± 0	5.5 ± 0
	Contact	5.5 ± 0	4.75 ± 0.66	5 ± 0.66	4.83 ± 0.63	5.25 ± 0.43	5.0 ± 0.43
Control		0	0	0	0	0	0

a *Six SPF chickens were inoculated intranasally (i.n.) with 10^3^ EID_50_ of virus in a 0.1 ml volume in G2 and G3 group, and three naive contact chickens housed with them, respectively; on 3 DPI, three inoculated and all dead naive chickens in each group were euthanized, and virus titers were determined in samples of heart, liver, spleen, lungs, kidneys, and brain in eggs*.

b *For statistical analysis, a value of 1.5 was assigned if the virus was not detected from the undiluted sample in three embryonated hen eggs (Sun et al., 2011). Virus titers are expressed as means ± standard deviation in log10EID_50_/0.1 ml of tissue*.

In the G2 group, virus shedding was detected from the oropharynx and cloaca swabs in inoculated chickens within seven DPI. The virus titers from oropharynx swabs and cloacal swabs were 2.92 and 1.96 log EID_50_, 2.63 and 1.88 log EID_50_, and 2.63 and 1.63 log EID_50_ on three DPI, five DPI, and seven DPI, respectively (Table 4). All of the chickens in the G2 group died within eight DPI. G3 virus shedding could be tested from both oropharyngeal and cloacal swabs in inoculated chickens on three DPI, and the virus titers were all 4.5 logEID_50_ (Table 4). All of the chickens in the G3 group died within four DPI. These showed that the duration of virus shedding of chickens infected with G2 was 8 days and was longer than the 4 days of G3, but the titers of replication of G2 group were lower than that of G3. Therefore, our results indicated that both G2 and G3 were highly pathogenic to chickens, and the replication of the G3 virus was higher than that of the G2.

**Table 4:** **Virus titers in oropharyngeal and cloacal swabs from chickens after inoculated with the two H5N1 HPAIVs (A/Chicken/China/G2/2012(H5N1) = G2 and A/Duck/China/G3/2012(H5N1) = G3)**.

**Strains**		**Days post-inoculation (log**_**10**_**EID**^**50**^**/0.1 ml)** ± **SD**^**a**^							
		**3 day**		**5 day**		**7 day**		**9 day**	
		**Oropharyngeal swabs**	**Cloacal swabs**	**Oropharyngeal swabs**	**Cloacal swabs**	**Oropharyngeal swabs**	**Cloacal swabs**	**Oropharyngeal swabs**	**Cloacal swabs**
G2	Infected^b^	2.92 ± 1.11 (5/6)	1.96 ± 0.46 (5/6)	2.63 ± 0.18 (2/2)	1.88 ± 0.53 (1/2)	2.63 ± 0.18 (2/2)	1.63 ± 0.18 (1/2)	ND^d^	ND
	Contact^c^	0	0	0	0	0	0	0	0
G3	Infected	4.5 ± 0(1/1)	4.5 ± 0(1/1)	ND	–	–	–	–	–
	Contact	4.5 ± 0(2/2)	4.5 ± 0(2/2)	ND	–	–	–	–	–
Control		0	0	0	0	0	0	0	0

a *For statistical purposes, a value of 1.5 was assigned if virus was not detected from the undiluted sample in three embryonated hen's eggs (Sun et al., 2011)*.

b *Chickens inoculated with virus*.

c *Contact chickens housed with those inoculated*.

d *ND: not detected. Chickens all died*.

### Transmission of H5N1 HPAIVs in chickens

To understand the naive contact transmission of these two viruses, three SPF chickens were inoculated intranasally with 0.1 ml PBS as naive control group and housed with inoculated chickens of the G2 and G3 groups, respectively. Oropharyngeal and cloacal swabs were collected from them at 3, 5, 7, 9, and 11 DPI. All surviving chickens were observed for 14 days. We collected and titrated the tissues and swabs for virus infectivity.

During the observation period, the naive contact chickens in the G2 group began to show mild clinical signs, such as spirits atrophy and inappetence, by five DPI and these mild clinical signs disappeared by seven DPI, all chickens survived for 14 days and the seroconversion rate was 100%. The naive contact chickens in the G3 group began to show clinical signs by two DPI and all died by four DPI (Figure 2). In the G3 naive contact group, virus replication titers were 5.5 log EID_50_ in the heart, 4.75 log EID_50_ in the liver, 5 log EID_50_ in the spleen, 4.83 log EID_50_ in the lungs, 5.25 log EID_50_ in the kidneys, and 5 log EID_50_ in the brain (Table 3). However, no virus was detected in tissue samples of the G2 naive contact group. All of the naive contact chickens in G3 group could shed virus from the oropharynx and cloaca swabs; the virus titers were both 4.5 log EID_50_ at three DPI, which was the same as that of inoculated chickens (Table 4). In the chickens of the G2 naive contact group, virus shedding could not be detected all along (Table 4). These showed that the lethality of naive contact chickens of G3 was 100% and that of G2 was 0 although both G2 and G3 had naive contact transmission in chickens (Table 2). So G3 virus had stronger transmissibility between chickens by naive contact than G2.

### Antigenic variation of HPAIV and protective efficacy of current commercial vaccines

To characterize antigenic variation of the two H5N1 HPAIVs and the current commercially vaccines strains, we carried out the cross reactive HI assay. The cross reactive HI antibody titers of anti-Re-4, anti-Re-6, and anti-D7 serum in reaction with G2 antigen were 5 log2, 1 log2, and 2 log2, respectively; and those in reaction with G3 antigen were 5 log2, 6 log2, and 9 log2, respectively (Table 5). Our results showed that the antigens of G2 and G3 were very different from those of vaccine isolates. Therefore, we should assess the immunogenicity and effectiveness of three current commercially inactivated vaccines against these isolates in 2012 in Guangdong of China.

**Table 5:** **Cross reactive hemagglutination inhibition (HI)^a^ antibody titers of anti-serum against five avian influenza virus antigens**.

**Strains**	**Clade**	**Serum of HI titers (log2)**				
		**Re-4**	**Re-6**	**D7**	**G2**	**G3**
Re-4	7	10	3	8	2	5
Re-6	2.3.2	5	8	10	1	10
D7	2.3.2	5	6	9	1	9
G2	7.2	5	1	2	8	5
G3	2.3.2.1	5	6	9	0	10

a *The cross reactive HI assays were carried out according to WHO standard method*.

Because H5N1 HPAIVs of clade 2.3.2.1 have most isolates from Guangdong in 2012, we estimated the effectiveness of current commercial vaccines against G3. Three-week-old SPF chickens were immunized with inactivated vaccines such as Re-4, Re-6, and D7, respectively. Serum from every group was collected at 14 and 28 DPV for HI test, respectively. Then, all chickens at 28 DPV were challenged intranasally with 200 μl 10^6^ EID_50_ of G3. In the Re-4 group, chickens were challenged with G3 when the mean HI titer was 9.4 log2 at 28 DPV. The chickens began to show clinical symptoms on three DPI and die on six DPI, shedding virus was tested on three to nine DPI, and the mortality and virus shedding proportion were 33.3 and 88.9%, respectively (Table 6). In the Re-6 group, chickens were challenged when the mean HI titer was 7.0 log2 at 28 DPV. The chickens began to die on six DPI, virus shedding was detected on three to eleven DPI, and the mortality and virus shedding proportion were 11.1 and 44.4%, respectively (Table 6). Those results showed that the Re-6 vaccine has a certain degree of protection against the G3 virus. In the D7 group, chickens were challenged when the mean HI titer was 7.4 log2 at 28 DPV, and no virus shedding or death was found during the observation period (Table 6). In the non-immunized control group, the HI titer was zero. All chickens were detected virus shedding and died during the observation period. These findings showed that the protection rate of the D7 vaccine against G3 was 100%, that of Re-6 was 88.9%, and Re-4 was 66.7%.

**Table 6:** **Results of hemagglutination inhibition (HI) titers from serum samples of chickens at 28 DPV. And the protection rates of three vaccines against G3 virus challanged^a^**.

**Groups**	**Before challenged**	**Post-challenge**	**Survival rate (%)**
	**HI antibody titers^b^ (log2)**	**Viruses shedding (%)**	
Re-4	9.40^c^	88.9	66.7
Re-6	7.20	44.4	88.9
D7	7.50	0	100
Control	0	100	0

a *Thirty-six three-week-old SPF chickens were divided into four groups and immunized with inactivated Re-4, Re-6, D7, and PBS respectively. At 28 DPV, all chickens were challenged intranasally with 200 ul 10^6^ EID_50_ of G3*.

b *Serum samples from Re-4 group, Re-6 group, and D7 group were detected with Re-4, Re-6, and D7 inactivated antigens, respectively. Serum samples from control group were detected simultaneously with Re-4, Re-6, and D7 inactivated antigens*.

c *Geometric mean titer (GMT)*.

In a word, the mean HI titers in all immune groups were higher than 6 log2, which indicated that these three commercial vaccines (Re-4, Re-6, and D7) had good immunogenicity in chickens. And the results of challenge study showed that these vaccines gave certain protection against G3, but their protection rates were different. Combined with the results of the cross reactive HI assay, we found that some vaccine strains were not antigenically well-matched with epidemic isolates, so the protective effects of the three vaccines varied.

## Discussion

The first H5N1 HPAIV in China was isolated from sick geese in Guangdong province in 1996 (Xu et al., 1999). In the following years, H5N1 HPAIVs repeatedly caused serious outbreaks in South China, especially in Hong Kong, and resulted in heavy losses of economy and life. Most of H5N1 viruses rapidly spread and induced large numbers of death within 2 or 3 days in chickens. The ducks and geese infected H5N1 HPAIVs showed no clinical symptoms in the past, but new H5N1 HPAIVs could attacked ducks and/or geese and caused deaths in recent years (Li et al., 2010). In addition, more and more mammal was susceptible to H5N1 HPAIV by natural or laboratory infections. Felines, including cat, tiger, lion, leopard, clouded leopard, and Asiatic golden cat were highly susceptible to H5N1 HPAIV (Reperant et al., 2009). The domestic dog, hamster, rhesus macaque, cynomolgus, palm civet, red fox and raccoon could be potentially fatal by H5N1 HPAIV. Pika, domesticated swine, cattle, donkey, rat, and rabbit can exhibit asymptomatic or nonfatal infections by H5N1 HPAIV (U.S. Geological Survey, 2011). From 2010 to 2013 the dominant clades of H5N1 HPAIVs co-circulating in South China were 2.3.2.1 and 7.2 although other clades, such as 2.3.4, had occasionally been detected (World Health Organization, 2013a). In our study, the G2 and G3 strains from poultry in Guangdong in 2012 belonged to clades 7.2 and 2.3.2.1, respectively. The inoculation dose was mostly 10^6^ EID_50_ or 10^5^ EID_50_ in previous pathogenicity studies, but here we selected a medium infective dose (10^3^ EID_50_) to observe the difference of pathogenicity and transmission of the H5N1 HPAIVs. This might be one of the reasons why the naive contact chickens in G2 group showed mild clinical symptoms without any viruses shedding or death. In our study, both G2 and G3 virus could highly replicated in the heart, liver, brain, spleen, kidneys, and lungs of infected chickens, virus shedding could be detected from all infected chickens during survival, and the lethal rates were both 100%. These results showed that G2 and G3 virus had high pathogenicity to chickens. By this token, the new H5N1 HPAIVs of these two clades in South China still had high pathogenicity to chickens.

AIV could form an aerosol and horizontal transmission through the respiratory tract in poultry. In recent years, the dominant AIVs co-circulated in mainland China were H5 and H9 subtypes, of which H9 subtype AIVs have strong horizontal transmission. However, only some H5 AIVs had horizontal transmission ability (World Health Organization, 2013a). In our previous studies, some H5N1 AIVs, which belonged to clades 0, 2.3.2.2, 7.2, and 9, could horizontally transmit between chickens, ducks, geese, Japanese quails, and mice (Sun et al., 2011). Here, G2 and G3 virus belonged to clades 7.2 and 2.3.2.1, respectively, and both of them could transmit horizontally in chickens. All of the naive contact chickens in the G3 group had detected viruses shedding and replication in organs, but the naive contact chickens in the G2 group only had mild clinical symptoms and had no death or virus shedding. These results showed the new H5N1 HPAIVs of these two clades had different horizontal transmission ability. Moreover, H5N1 HPAIV of clade 2.3.2.1 still have been circulating in poultry and wild bird up to now, so they will continue to have the threat to human health and poultry product.

In China, poultry production modes, including rural household scatter breeding, poultry farms, and modern poultry ranches, are multiple and complicated so that prevention and control of H5 HPAI are difficult. Therefore, all poultry in China were immunized with H5N1 vaccinations compulsorily. In recent decades, several H5 vaccines, especially H5 inactivated vaccines, were widely used in China due to constant mutation and evolution of the virus (Swayne, 2012). The first commercial flu vaccine in China was an H5N2 inactivated vaccine, which used the low pathogenic avian influenza H5N2 virus A/Turkey/England/N-28/1973 and was approved for use in August of 2003 (Chen and Bu, 2009). Then the Re-1 vaccine was approved for use in 2004, for which was antigenically well-matched the epidemic strains at that time. In 2006, the H5N1 Re-4 vaccine, whose strain belonged to clade 7, was approved into service in China and widely used in the northern mainland. In 2008, new Re-5 vaccine began to be used in northern and southern China, whose strain A/duck/Anhui/1/2006(H5N1) belonged to clade 2.3.4 (Jiang et al., 2010; Li et al., 2010). In 2012, the recombinant vaccine Re-6 was also approved for use in the mainland to control new epidemic strains in clade 2.3.2. In 2013, a new H5 vaccine D7 was approved for use in waterfowl, which used an H5N2 virus (A/duck/Guangdong/D7/2007) belonged to clade 2.3.2. In conclusion, although flu vaccines were updated constantly, new strains still continue to appear in China. Therefore, it is necessary to evaluate the effectiveness and effects of current vaccines against the new strains timely.

From 2011 to 2012, most H5 isolates circulating in Guangdong province belonged to clade 2.3.2.1, including G3, so we wanted to estimate the effectiveness of current vaccines against them. In our study, the effectiveness of these three commercial vaccines against G3 varied. The D7 vaccine provided 100% protection to chickens against G3, the Re-6 vaccine provided 88.9% protection, and the Re-4 vaccine only provided 66.7%. The antibody titer of Re-4 in chicken had more 2 log2 than Re-6 and D7 when challenged at 28 DPV, but the protection rate of Re-4 against G3 was lowest because the Re-4 vaccine strain and G3 belonged to different clades. These results indicated that Re-4 vaccine did not protect chickens against H5 viruses challenging although had good immunogenicity and could induce high antibody levels. The D7 vaccine provided the best protection in these three vaccines against G3, whose strain belonged to clade 2.3.2. These told us that high antibody levels did not provide good protection, what need antigen matching between vaccine and epidemic strains. Therefore, to evaluate vaccines more objectively and effectively, we should be concerned not only about antibody level of immunized animals but also antigen matching between vaccine strains and epidemic isolates when observed the protection of vaccines in clinical practices.

## Author contributions

Conceived and designed the experiments: PJ, ML. Performed the experiments: XL, HS, YS, JC, NQ. Analyzed the data: PJ, HS, XL. Contributed reagents/materials/analysis tools: PJ, XL, YS, JC, JY, SW, NQ, TZ. Wrote the paper: HS, PJ. All authors read and approved the final manuscript.

### Conflict of interest statement

The authors declare that the research was conducted in the absence of any commercial or financial relationships that could be construed as a potential conflict of interest.

## Footnotes

^1^Food and Agricultural Organization(FAO). H5N1 HPAI Global Overview - July and August 2010, prepared by EMPRESS/GLEW, Issues No. 24.
